# Heart Team for Left Appendage Occlusion without the Use of Antithrombotic Therapy: The Epicardial Perspective

**DOI:** 10.3390/jcm11216492

**Published:** 2022-11-01

**Authors:** Stefano Branzoli, Fabrizio Guarracini, Massimiliano Marini, Giovanni D’Onghia, Domenico Catanzariti, Elettra Merola, Luciano Annicchiarico, Giulia Casagranda, Chiara Stegagno, Mauro Fantinel, Mark La Meir

**Affiliations:** 1 Department of Cardiac Surgery, UZ Brussel, 1050 Brussels, Belgium; 2 Cardiac Surgery Unit, Santa Chiara Hospital, 38122 Trento, Italy; 3 Department of Cardiology, Santa Chiara Hospital, 38122 Trento, Italy; 4 Heart Rhythm Management Centre, UZ Brussel, 1050 Brussel, Belgium; 5 Cardiology Unit, Santa Maria del Carmine, 38068 Rovereto, Italy; 6 Gastroenterology Unit, Santa Chiara Hospital, 38122 Trento, Italy; 7 Neurosurgery Unit, Santa Chiara Hospital, 38122 Trento, Italy; 8 Department of Radiology, Santa Chiara Hospital, 38122 Trento, Italy; 9 Neurology Rehabilitation Unit, Eremo Hospital, 38122 Trento, Italy; 10 Cardiology Unit, Santa Maria Hospital, 32032 Feltre, Italy

**Keywords:** epicardial appendage occlusion, thoracoscopic surgery, stroke prevention, hemorrhage prevention, atrial fibrillation, heart team

## Abstract

Background: Left atrial appendage occlusion is an increasingly proposed treatment for patients with atrial fibrillation and poor tolerance to anticoagulants. All endovascular devices require antithrombotic therapy. Anatomical and clinical variables predisposing to device-related thrombosis, as well as post-procedural peri-device leaks, could mandate the continuation or reintroduction of aggressive antithrombotic treatment. Because of the absence of foreign material inside the heart, epicardial appendage closure possibly does not necessitate antithrombotic therapy, but data of large series are missing. Methods: Multidisciplinary team evaluation for standalone totally thoracoscopic epicardial appendage closure was done in 180 consecutive patients with atrial fibrillation and poor tolerance to antithrombotic therapy. One hundred and fifty-two patients consented (male 66.1%, mean age 76.1 ± 7.4, CHA_2_DS_2_VASc mean 5.3 ± 1.6, HASBLED mean 3.8 ± 1.1). Indications were cerebral hemorrhage (48%), gastro-intestinal bleeding (33.3%), and other bleeding (20.7%). No antithrombotic therapy was prescribed from the day of surgery to the latest follow up. Results: Procedural success was 98.7%. At a mean follow up of 38.2 ± 18.8 months, cardioembolic and bleeding events were 1.3% and 0.6%, respectively. Among patients with a history of blood transfusions (41.1%), none needed further transfusions or treatment post procedure. Conclusion: Epicardial appendage occlusion without any antithrombotic therapy appears to be safe and effective. This strategy could be advised when minimization of bleeding risk concomitant to stroke prevention is needed.

## 1. Introduction

Cerebrovascular events are the third leading cause of death in developed countries. Atrial fibrillation (AF) accounts for 15–30% of these events and requires stroke prevention treatment options with new oral anticoagulants (NOACs) as first line therapy over Vit K antagonists (OACs) as a pharmacological strategy [1]. Despite guidelines, anticoagulants are not prescribed in up to 30% of AF patients (ORBIT-GAREFIELD registry) [2]. According to the ROCKET, ARISTOTLE, and RE-LY studies, the prescription of NOACs is associated with a discontinuation rate of 23.7%, 25.3, and 20.7%; 1.7%, 1.2%, and 1.5% risk of stroke/year; and 14.9%, 18.1%, and 14.6% risk of bleeding/year, with a rate of major hemorrhage of 3.6%, 2.1%, and 2.7%, respectively [3,4]. To improve bleeding outcomes, lower dose NOACs regimens have been tested. A meta-analysis of randomized trials of patients on low dose NOACs vs. warfarin has shown similar overall reductions in cardioembolism, and a non-significant reduction in major bleeding with a significant reduction in intracranial bleeding [5]. As bleeding is a major cause of NOACs/OACs discontinuation, left atrial appendage occlusion (LAAO) has become an interventional therapeutic option [1]. PROTECT-AF, PREVAIL, and PRAGUE-17 trials have shown potential and drawbacks, mainly bleeding, of LAAO with the mandatory post procedural antithrombotic therapy [5,6,7]. The findings of the PRAGUE-17 trial with those of AVERROES support the idea that higher bleeding rates are expected in higher HASBLED risk profile cohorts on single antiplatelt therapy (SAPT) [7]. A meta-analysis of all of the major trials has demonstrated no statistically significant reduction in major bleeding of LAAO compared with (N)OAC [8]. Ewolution and Aplatzer-Amulet’s studies, reporting excellent success rates of implantation, and trend towards less aggressive post procedure antithrombotic therapy to improve bleeding outcomes, have shed further light on the potentials of interventional strategies for stroke prevention in AF [9,10]. However, bleeding remains an issue with any antithrombotic therapy according to ACTIVE and ASPREE trials [11,12]. In addition, the increasing number of procedures have brought to attention peri-device leak (PDL) and device-related thrombosis (DRT), both requiring the reintroduction of antithrombotic treatment, re-presenting the dilemma of an appropriate pharmacological regimen in patients referred to LAAO because of a poor tolerance to antithrombotic therapy [13,14,15]. Epicardial LAAO because of the absence of a foreign material inside the heart potentially does not require any antithrombotic therapy, addressing some anatomical, procedural, and clinical issues improving bleeding outcomes. Only few reports with this approach are published and all have a post procedure period of antithrombotic therapy [16,17,18,19]. Here, we describe our experience regarding the safety and efficacy of standalone totally thoracoscopic epicardial appendage clipping with immediate antithrombotic discontinuation.

## 2. Materials and Methods

### 2.1. Patients Selection

The inclusion criteria were a diagnosis of any type of atrial fibrillation, CHA_2_DS_2_VASc > 2, HAS BLED > 1, or poor tolerance to OAC/NOAC, defined as at least one of the following: life threatening hemorrhage, more than two hospital admissions for bleedings on (N)OACs requiring treatment, hemoglobin decrease > 2 g/dL, chronic underlying disease predisposing to a high risk of rebleeding if on APT. A multidisciplinary team (an electrophysiologist, a cardiac surgeon, an anesthesiologist, a neurologist, a gastroenterologist, and the referring physician) evaluated all patients. Exclusion criteria were life expectancy <1 year, ongoing DAPT for recent PTCA, contraindication to single right lung ventilation, concomitant cardiac surgery, indication to AF ablation (Table 1). All patients requiring appendage occlusion at our institution were discussed by the Heart Team and the criteria of this study were additional to the flow-chart accepted by all members of the team, which were previously published [20]. The preoperative workup included heart CT scan and echocardiogram for potential coronary artery disease/structural heart disease requiring treatment, and carotid doppler ultrasound for baseline neurologic follow up. As none of the patients had signs or symptoms of lower limb ischemia, doppler ultrasound for POVD was not routinely performed.

### 2.2. LAAO-T Procedure

The procedure was performed as described previously [21]. Briefly, three ports in a “hockey stick” configuration were introduced in the left hemithorax between the anterior and mid-axillary line in the III, V, and VII intercostal space. After CO_2_ insufflation, the pericardium was opened, the LAA was measured, and the AtriClipPro2 (AtriCure Inc., Mason, OH, USA) was deployed at its base under direct view and TOE guidance.

### 2.3. Post Procedure Pharmacological Therapy

No antithrombotic therapy was prescribed from the day of surgery to the latest follow up, except in four patients requiring SAPT for atherosclerotic disease.

Of these four patients, two had a previous stroke, and the others, despite the degree of carotid artery stenosis, received no antiplatelet therapy, because in presence of asymptomatic carotid stenosis, antiplatelet therapy is not mandatory according to the guidelines of the European Society of Vascular Surgery (class of indication IIa level of evidence C), and in the US version no recommendation is given [22,23]. In all cases with carotid stenosis >50% the decision by the Heart Team regarding the appropriate antiplatelet regimen was always discussed with the patient. 

### 2.4. Follow up

All patients underwent outpatient clinic visit with physical examination, ECG, laboratory tests, and completion of the Questionnaire for Verifying Stroke Free Status (QVSFS) at 1, 2, 6, and 12 months and annually thereafter. This questionnaire, validated by the European Society of Neurology, was chosen as it has been shown to be effective at identifying stroke free individuals with accuracy, also in a population with a large proportion of patients with previous stroke or TIA. In the case of at least one positive answer, a CT scan was planned [24]. For appendage closure assessment, TOE and CT scans at 1–3 months were scheduled. Criteria of success was considered a stump less than 1 cm [25]. Although arbitrary, this value is widely accepted and has been shown to be a risk factor for DRT for endovascular devices [26].

### 2.5. Statistical Analysis

Categorical variables are expressed in numbers (n/N) and percentages (%). Continuous variables with a normal distribution using a Shapiro–Wilk test are expressed as mean ± standard deviation (SD), median, and interquartile range (Q1–Q3) when meaningful. The individual patient annual risk for stroke and bleeding was calculated in accordance with CHA_2_DS_2_VASc and HASBLED, and then calculated for the population (expected risk rate) and compared with the corresponding observed risk rate [27,28]. The relative risk reduction (RR) was then calculated as (observed risk − expected risk)/expected risk × 100. To analyze event rates at the latest follow up for cardiovascular death, cardioembolism, and hemorrhage, the Kaplan–Meier survival analysis was calculated. A Log rank test was performed for comparison.

## 3. Results

### 3.1. Patients

One-hundred and eighty consecutive patients underwent a multidisciplinary team evaluation: 152 (age 76.9 ± 6.7, male 65.8%, CHA_2_DS_2_VASc 5.4 ± 1.6, HASBLED 3.8 ± 1.1, cerebral hemorrhage 55.0%, previous cardioembolism 14.1%, GI bleeding 31.3%, non-cerebral/GI bleeding 15.3%, and anatomy unsuitable for percutaneous procedure 5.5%) underwent LAAOT. The patients with an unsuitable anatomy for the percutaneous procedure were not referred for ablation as this would have required at least three months of oral anticoagulation and this period was considered by the Heart Team as being at too high risk for bleeding for each of the patients. Twenty-eight patients (age 71.5 ± 9.4, male 83.3%, CHA_2_DS_2_Vasc 4.3 ± 1.1, HASBLED 3.7 ± 1.3, cerebral hemorrhage 40%, previous stroke 28%, GI bleeding 52%, and non-cerebral/GI bleeding 4%) denied consent (nLAAOT).

### 3.2. Procedural Outcomes

All but 2 of the 152 patients underwent LAAOT. Operative data and outcomes are shown in Table 2.

**Figure 1:**
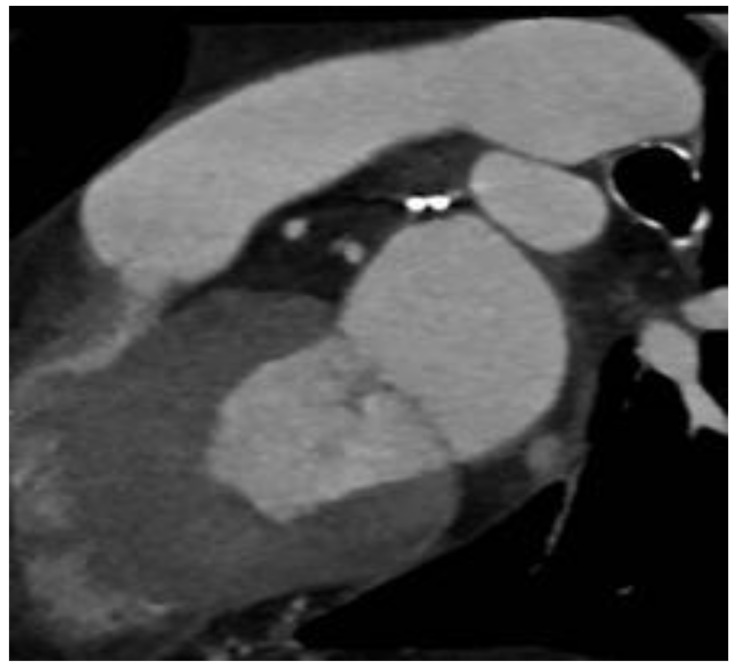
Clip short axis.

**Figure 2:**
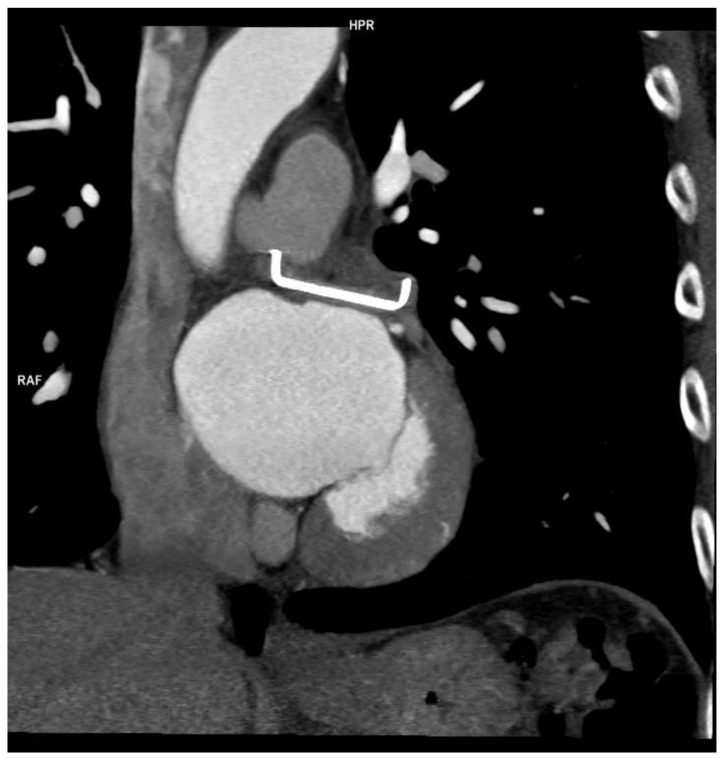
Clip long axis.

No deaths or conversion to thoracotomy or device-related complications were reported. Eighteen patients had their appendage successfully closed despite pericardial adhesions. For this reason, two were referred to percutaneous closure. The only complication reported was pericarditis in 7 of the first 50 patients, and none were reported after the introduction of prophylactic colchicine at 0.5 mg bid or indomethacin 50 mg bid. TOE and CT scans showed satisfactory device deployment in all patients, with a mean stump of 2.4 ± 2.3 mm and absence of stump in 81% of cases (Figure 1 and Figure 2).

### 3.3. Follow up

**Figure 3:**
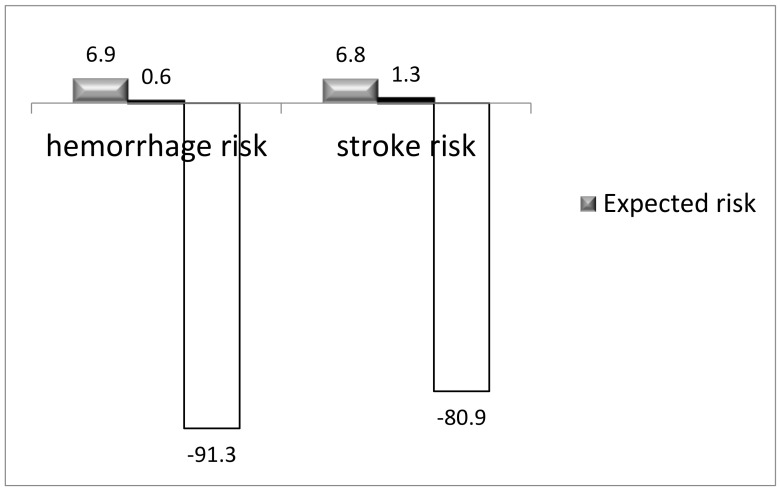
RR reduction for hemorrhage and stroke.

Clinical and imaging follow up for the appendage was complete for all 180 patients (mean 38.2 ± 18.8 months, range 65–6 months), resulting in 377.9 patient/year for stroke and 378.8 patient/year for hemorrhage. In the LAAO-T group, no hospital re-admissions were documented for cardiovascular events related to the procedure. All patients completed the QVSFS. There was one minor stroke and one TIA (10 and 5 months, respectively) in the absence of a significant LAA stump at CT scan. On the QVSFS, no suspicion of neurological events was raised in all other patients. The actual ischemic rate was 1.3% compared with the expected adjusted mean stroke rate of 6.8%/year. None of the patients (46.6%) in the LAAOT group with a history of repetitive blood transfusions were readmitted for blood requirement. With one cerebral hemorrhage (cerebral artero–venous malformation), the rate of bleeding was 0.6%, which compared favorably with the expected 6.9% predicted bleeds/years, but the effective reduction was higher, considering that 7.9% of patients had HASBLED > 5, and for this value, the effective bleeding risk/year is not available in literature (Figure 3) [27].

**Figure 4:**
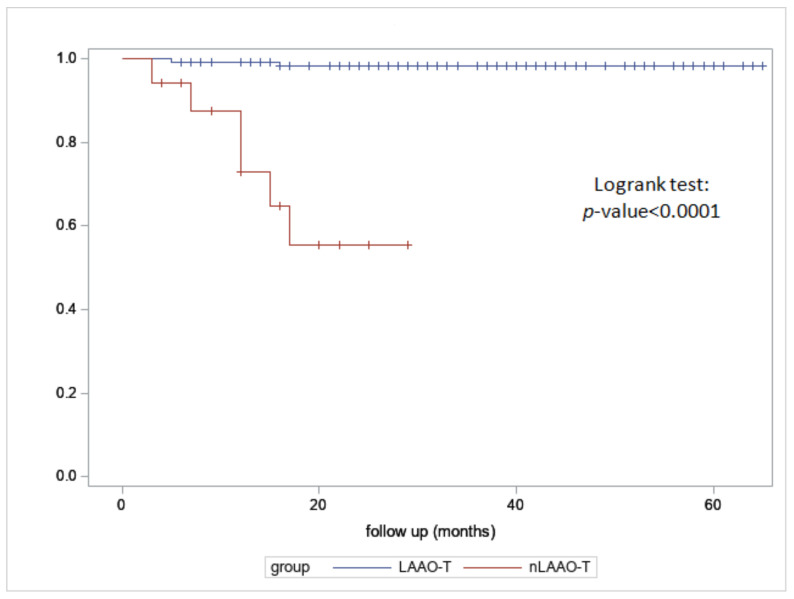
Kaplan–Meier for stroke for LAAO-T and nLAAOT.

**Figure 5:**
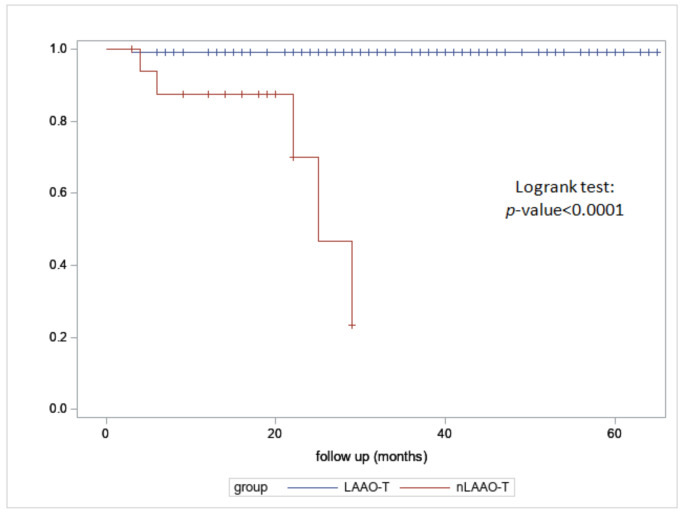
Kaplan–Meier for hemorrhage for LAAOT and nLAAOT.

In the LAAOT patients, there were four deaths at 50 days, 6 months, 1.5 year, and 1.9 years for the recurrence of cerebral hemorrhage, multi-organ failure, pneumonia, and SARS-CoV-2 infection, respectively. Of the 28 patients who denied consent (nLAAOT) (12 on LMWH and 16 on APT), 6 died (3 cerebral hemorrhage, 2 strokes, and 1 GI re bleeding), 4 experienced repetitive transfusion/treatment during follow up (total re bleeding event 28.5%), and 1 cardioembolic event (total cardioembolic event 10.7%), and the remainder experienced no adverse events (Figure 4 and Figure 5).

## 4. Discussion

Up to date, this is the first study on the safety and efficacy for haemorrhage and stroke prevention of an epicardial approach to LAAO in the absence of any antithrombotic therapy from the immediate postoperative day. Cardioembolism and hemorrhage are the two faces of the same coin when deciding upon optimal antithrombotic therapy in AF [1]. (N)OACs are first line therapy, but bleeding is a major cause of discontinuation [2,3,5]. Antiplatelet therapy may only partially address cardioembolic issues, and bleeding remains a problem, as shown by the ACTIVE and ASPREE trials [11,12]. In the case of major bleeding complications, the decision when to resume or discontinue antithrombotic therapy in the long-term is still the subject of debates [26,27,28,29,30,31]. In a report of Redfors on patients off (N)OACs for at least one year due to contraindication, incidences of ischemic and hemorrhagic stroke were 12 and 20.3%, respectively, and both were strongly correlated [32]. According to the TREAT-AF study, resumption of antithrombotic therapy at 90 days following major bleeding is associated with significant increased risk of non-intracranial bleeding and a trend to a lower risk of stroke, suggesting that stroke prevention strategies after major bleeding events could be beneficial if bleeding risk is minimized [33]. As the majority of thrombi in patients with AF are thought to come from the LAA [34], different devices for LAAO have been designed. The annual ischemic strokes rates with NOACs are 1–2%, and with percutaneous devices they range from 0 to 2.2% [8,9,10,35]. Meta-analysis of RCTs have shown a similar rate of ischemic stroke for percutaneous LAAO compared with N(OACs), and reductions in hemorrhagic strokes, but no statistically significant reduction in major bleeding [8]. Another meta-analysis of RCTs and 27 observational studies comparing major bleeding events in percutaneous LAAO vs. NOAC showed 2.2 events per 100 patient-year events vs. 2.5 events per 100 patient-year event, respectively [36].

Endocardial devices share four scenarios: immediate antithrombotic therapy, DRT, PDL, and a favorable LAA anatomy [35]. The PROTECT, PRAVAIL, and PRAGUE-17 trials, and EWOLUTION and AMPLATZER-AMULET studies have reported the potentials and drawbacks of endocardial devices [6,7,9,10]. In the EWOLUTION study, good implantation rates have been reported with 2.2% of patients excluded for suboptimal anatomy. The incidence of DRT was 4.1%, and 4.6% had major bleeding with 27% of patients on OAC/NOAC, 60% on DAPT, 8% (off label implant) without APT, and 7% on SAPT at discharge. At 2 years, 8% were still on OAC/NOAC, 7% on DAPT, 71% on SAPT, and 14% were without any antithrombotic therapy [9]. The AMPLATZER-AMULET study reported comparable procedural success, with incidence of DRT of 1.69%, with 57.7% of patients discharged on DAPT, 22.4% on SAPT, 11.2% on OAC, with an annual ischemic stroke rate of 2.2%/year, annual TIAs of 1.0%/year, and annual rate of major bleeding of 7.2%, ranging from 10.1%/year at 1 year and 4%/year thereafter. At 2 years, 15.7% of patients were still on OAC/DAPT, 62.8% on SAPT, and 21.5% were without any APT [10]. The detection of DRT and PDL requires the reintroduction of aggressive antithrombotic therapy [14,23] as they are potentially associated with a higher risk of death and cardioembolism [14,26,35,37].

DAPT with clopidrogel + aspirin, the most frequently used combination, increases the risk of major bleeding by up to 30% compared with SAPT alone [29]. In addition, this mandatory post procedure therapy may be not ideal for patients with high HASBLED or comorbidity with persistent high risk of bleeding on APT (i.e., degenerative amyloid angiopathy, Rendu Osler Weber syndrome, intracranial hemorrhages requiring prolonged follow up, some hematologic diseases, and GI angiodysplasia). To improve bleeding outcomes, a shortened post procedure period of NOAC/OAC/APT, despite a lack of large trials, has been suggested [9,10]. All of the above suggest a clinical need to further optimize bleeding outcomes in frail patients. The 2020 ESC guidelines state that for patients who do not tolerate any antiplatelet therapy, either an epicardial catheter approach (e.g., Lariat system) or thoracoscopic clipping of the LAA may be considered. Based on this evidence, we designed a study whose peculiarities are as follows: a high CHA_2_DS_2_VASc and HASBLED score, based on the Heart Team assessment and on immediate antithrombotic therapy discontinuation post implantation.

In our experience, LAAO thoracoscopically was successfully performed in 98.7% of cases. This is in line with other papers on epicardial and endocardial devices for LAAO [9,10,16,17,18,19]. However, with percutaneous devices, 15–17% of successfully implanted patients are still on (N)OAC/DAPT after 2 years [9,10]. Therefore, although LAAO may be occasionally voluntarily associated with NOACs prescription, the procedural success does not always reflect treatment success. This is further affected by the incidence of DRT and PDL requiring the reintroduction of antithrombotic therapy [13,14,15,29]. In our experience, a successful procedure equals treatment success, as no patient received any antithrombotic therapy from surgery to the latest follow up. This compares favorably to all up to date published reports on epicardial devices all including a period of APT and with the largest up to date paper on LAAO with Lariat with 98% effective complete closure [15,16,17,18].

The clipping device used has been specifically designed for LAAO in contrast with endostaplers, which have a lower success outcome, as reported by Lee et al. [37]. Delivering the clip with a simplified and standardized thoracoscopic technique requiring only basic thoracoscopic skills may be interesting for centers with larger volumes of LAAO procedures, as an epicardial approach could be the solution for those technically ineligible for a percutaneous procedure [7,8,9,10,13,15,18,19].

Pericarditis was the only complication reported and the introduction of colchicine dramatically reduced the incidence of tissue inflammation, contributing to no further events. This is in accordance with Gunda et al. [38] and compares favorably to other epicardial procedures with 8.3−14% incidence of pericarditis [18,19,35].

No anatomically ineligible appendages were found and all patients ineligible for the percutaneous device were successfully treated thoracoscopically. The absence of stump was reported in 81% of our series, in line with Caliskan et al. with 72% of no stump in the open chest surgery, and suggests that a thoracoscopic approach may provide results comparable to open chest surgery [39]. Therefore, data on the efficacy of this device can probably be mutually extrapolated from both accesses. The lariat is the only other available epicardial device specifically designed for LAAO, and in all reports published, adhesions have been described as a contraindication in all cases [40,41]. The success of implantation in our experience in the case of adhesions led us to the conclusion that adhesions should not be considered a contraindication, but detailed discussion with patients is mandatory to clarify the potential risks of the thoracoscopic procedure. For those eligible patients who denied consent to the procedure, self-perception of their frailty was the leading reason for refusal. The incidence of cardioembolism (1.3 vs. 10.7%) and hemorrhage (0.6 vs. 28.5%) of LAAOT vs. n-LAAOT patients is in line with the only up to date published paper of Parikh et al. [19], with comparable HASBLED to ours, reporting on 108 lariat patients vs. 45 patients excluded from LAAO (cardioembolism 1.9% vs. 24%, hemorrhage 9.2 vs. 24.4%), suggesting that in patients with high HASBLED, epicardial LAAO seems to have a better prognosis than the resumption of subtherapeutic antithrombotic therapy [19,32,33].

In terms of cardioembolism prevention with a mean CHA_2_DS_2_VASc of 5.3 ± 1.6 for this study, a 80.9% relative risk reduction for stroke in the absence of any antithrombotic therapy at more than 3 years is in line with the reported 63−86% of percutaneous studies, all with a lower mean CHA_2_DS_2_VASc [9,10] and with the results reported in a multicenter study on epicardial appendage clipping [17].

However, in this latter multicenter study, the stroke preventive effect of the post procedure antithrombotic therapy at discharge ((N)OAC 10%, 3% LMWH, 41% on SAPT, and 2% on DAPT) and during follow up not objectively specified might have influenced the results in the median follow up of 12.5 months [17]. The results by Litwinowicz et al. are promising [41] for long term outcomes with the Lariat, with a mean follow up of 4.2 years, documenting 81% risk reduction for stroke and 78% risk reduction of bleeding, but the post procedural antithrombotic therapy (58% on anticoagulants) again might have had an impact on the event rate calculations. These findings seem to be confirmed by the long term clinical outcomes for lariat reported by Parikh et al., with a systemic thromboembolic event rate of 1.9% at a follow up of 6.5 ± 0.8 years, 15.7% without any antiplatelet therapy from the third month [19], and in the largest up to date European experience with Lariat by Tilz et al., with 1.8% incidence of cardioembolic events at a mean follow up of 181 ± 72 days with post procedure antiplatelet therapy [42,43].

Although promising, all these data do not allow for drawing definitive conclusions on the effective added value of an epicardial closure whose peculiarity is the absence of the required post procedure APT. This might only help the up to date report of epicardial LAAO with the shortest period of anticoagulation and no further antithrombotic therapy by Ohtsuka et al. documenting two cardioembolic events and no bleedings on 201 patients at a mean follow up period of 48 months [18].

In terms of bleeding outcomes, considering the mean HASBLED and the distribution of our population, a 88.9% relative risk reduction compared favorably with all of the up to date published data on endovascular devices, all of which included the overall lower predicted risk of bleeding, and suggest that the overall 46% relative risk reduction of major bleeding reported by Boersma et al. and the 7.2% risk of hemorrhage by Hildick-Smith might be improved by the absence of antithrombotic therapy [9,10,11,31].

Patients with specific diseases with intrinsic high re-bleeding risk on antithrombotic therapy should preferably be treated with a solution that allows for immediate antithrombotic therapy discontinuation. In our series, except for one case of cerebral hemorrhage, no recurrence of bleeding was reported. Among the enrolled patients with repetitive bleeding requiring treatment, 33.3% had previous GI bleeding. After LAAOT, no further hospitalizations for transfusions were documented. This finding compares favorably with the only up to date report on a sub-category of 151 patients with previous GI bleeding who had undergone endocardial LAAO and subsequent antithrombotic therapy. In this report, 4% vs. 0.8% of major periprocedural bleeding were calculated among patients with previous GI bleeding and non-GI, respectively, with overall 4.6% vs. 1.5% major bleeding events at a follow up of 1.3 years and a 20.1% relative reduction according to the expected rate based on the HASBLED for GI group [44]. The comparison was also favorable with Hildick-Smith et al., reporting on 1088 patients implanted with Amplatzer Amulet: incidence of bleeding was 10.2%, mainly GI and prescription of a less aggressive antithrombotic therapy showed no significant difference between DAPT and SAPT (41.8% vs. 34.4%) (10), as GI bleedings’ indication to LAAO are often associated with a high bleeding recurrence on APT [45,46]. We expect our result to be confirmed over time due to the stable closure rate of the device documented after 5 years [47].

From the first trials on LAAO, there has been a trend towards a higher degree of frailty for eligible patients and, to improve outcomes, as in other cardiovascular diseases, a multidisciplinary approach has been advocated for, along with studies on endocardial vs. epicardial devices [48,49]. In a report comparing Watchman vs. Lariat, incidence of leaks was 21% vs. 14%, of thrombus 3.7% vs. 1.4%, and of stroke was 1.3% vs. 1.1%. In a preliminary report comparing transcatheter and thoracoscopic approaches, both strategies seemed to be safe and effective [20].

In the case of poor tolerance to (N)OACS, the optimal therapeutic option is the subject of active debate, especially in frail patients and the selection of the appropriate treatment option for pharmacologic, endovascular, or epicardial should probably be based on absolute risk of stroke and bleeding recurrence for a given patient in a multidisciplinary approach.

## 5. Conclusions

Epicardial appendage closure without antithrombotic therapy appears to be safe and effective for hemorrhage and stroke prevention at the midterm. In the presence of anatomical and clinical aspects predisposing to suboptimal implantation or the recurrence of bleeding on APT, an epicardial approach should be considered. Further studies comparing epicardial vs. endocardial devices, preferably randomized, are needed with the main aim of improving patient selection, as specific categories of patients might benefit from a specific treatment option.

## 6. Limitations

The number of patients, length of follow up, and being a single center study are major limitations. Although totally thoracoscopic cardiac surgery is not widely diffused, this procedure requires only basic thoracoscopic skills and is thus easily accessible for those centers willing to offer an epicardial option when endovascular LAAO is not feasible. The definition used to define the success of implantation is arbitrary and requires further evaluation for clinically relevant implications. HAS BLED is a useful scoring tool, but it is imperfect. A cerebral MRI to assess microembolization might help to detect subclinical strokes, but this would require comparison with a control group to be meaningful; it is of note that this data are missing in the largest studies on percutaneous devices. The absence of a control group is also a limitation, but as data on the safety and efficacy of stand alone epicardial LAAO are few, the present study was designed as potentially preliminary to a RCT that will require a control group.

## Figures and Tables

**Table 1:** Patients characteristics.

Characteristics	(n/N,%)
Male gender (n/N)	119/180 (66.1%)
Age (Years) mean ± SD	76.1 ± 7.4
CHA_2_DS_2_VASc mean ± SD	5.3 ± 1.6
HASBLED mean ± SD	3.8 ± 1.1
<3 (%)	(12.4)
3–5(%)	(79.7)
>5 (%)	(7.9)
Ejection fraction mean (%) Ejection fraction <40% Renal function n/N Cr Cl< 30 n/N Cr Cl 30–50 n/N Dialysis n/N	51.7 ± 8.3 16/180 (8.8) 23/180 47/180 3/180
Cr Cl > 50	107/180
Carotid Doppler Ultrasound (stenosis%)	
bilateral <50	160/180
monolateral 50–70	16/180
at least one > 70	4/180
Previous SARS-CoV-2 n/N (%)	12/180 (6.6)
Previous cardiac surgery n/N (%)	4/180 (2.2)
Previous Stroke n/N (%)	71/180 (40)
Previous stroke in (N)OACs	20/180 (11.1)
Cerebral Hemorrhage n/N (%)	84/180 (46.6)
Subdural n/N (%) Intraparenchimal n/N (%) Deg Cerebral Amyloid n/N (%) cAVM n/ N(%) Angiomas/cavernomas n/N (%)	25 (13.8) 20 (11.1) 19 (10.5) 13 (7.2) 4 (2.2)
Aneurisms	5 (2.7)
Miscellaneous	27/180 (15)
Hemathologic disease	11 (6.2)
Rendu Osler Weber syndrome	7 (4)
Others	9 (3.4)
GI Bleeding n/N (%)	60/180 (33.3)
Anatomy unsuitable for endovascular device n/N (%)	10/180 (5.5)
Total	180

Cr Cl:creatinine clearence; cAVM:cerebral arteriovenous malformation; GI:gastrointestinal.

**Table 2:** Intra-post operative data, pharmacological therapy.

Variable	
Duration of operation (min) m ± SD	30.7 ± 16.6
Hospital stay (days) m ± SD	3.2 ± 0.9
Post procedure antithrombotic therapy n/N	
SAPT	4/150
None	146/150
Pericarditis	7/150

## Data Availability

All of the data are available upon written request to the corresponding author.
